# Antimicrobial Peptides as Potential Anti-Tubercular Leads: A Concise Review

**DOI:** 10.3390/ph14040323

**Published:** 2021-04-02

**Authors:** Gabriel S. Oliveira, Raquel P. Costa, Paula Gomes, Maria Salomé Gomes, Tânia Silva, Cátia Teixeira

**Affiliations:** 1 ICBAS—Instituto de Ciências Biomédicas Abel Salazar, Universidade do Porto, P-4050-313 Porto, Portugal; jorge.oliveira@ibmc.up.pt (G.S.O.); sgomes@ibmc.up.pt (M.S.G.); 2 I3S—Instituto de Investigação e Inovação em Saúde, Universidade do Porto, P-4200-135 Porto, Portugal; 3 IBMC—Instituto de Biologia Molecular e Celular, Universidade do Porto, P-4200-135 Porto, Portugal; 4 LAQV-REQUIMTE—Departamento de Química e Bioquímica, Faculdade de Ciências da Universidade do Porto, P-4169-007 Porto, Portugal; pgomes@fc.up.pt; 5 LAQV-REQUIMTE—Departamento de Ciências Químicas, Faculdade de Farmácia, Universidade do Porto, P-4050-313 Porto, Portugal; raquel.piinho@gmail.com

**Keywords:** antimicrobial peptides, anti-tubercular drugs, antibiotic resistance, mycobacteria, tuberculosis

## Abstract

Despite being considered a public health emergency for the last 25 years, tuberculosis (TB) is still one of the deadliest infectious diseases, responsible for over a million deaths every year. The length and toxicity of available treatments and the increasing emergence of multidrug-resistant strains of *Mycobacterium tuberculosis* renders standard regimens increasingly inefficient and emphasizes the urgency to develop new approaches that are not only cost- and time-effective but also less toxic. Antimicrobial peptides (AMP) are small cationic and amphipathic molecules that play a vital role in the host immune system by acting as a first barrier against invading pathogens. The broad spectrum of properties that peptides possess make them one of the best possible alternatives for a new “post-antibiotic” era. In this context, research into AMP as potential anti-tubercular agents has been driven by the increasing danger revolving around the emergence of extremely-resistant strains, the innate resistance that mycobacteria possess and the low compliance of patients towards the toxic anti-TB treatments. In this review, we will focus on AMP from various sources, such as animal, non-animal and synthetic, with reported inhibitory activity towards *Mycobacterium tuberculosis*.

## 1. Tuberculosis: A Brief Overview

### 1.1. Global Burden

Tuberculosis (TB) is an airborne infectious disease caused by pathogens belonging to the *Mycobacterium tuberculosis* (*Mtb*) complex. Characterized by its high rate of both morbidity and mortality, it has been considered a public health emergency for the last 25 years and it is one of the deadliest of human infectious diseases [1]. The pathogenicity of *Mtb* is exacerbated when in the presence of the human immunodeficiency virus (HIV), as these pathogens can potentiate one another inside of an individual host [2]. Alongside *Plasmodium*, a genus of parasites responsible for malaria, these pathogens form the so-called “Big Three” infectious diseases [3]. According to the latest data from the World Health Organization (WHO), around 10 million people developed TB in 2019, with an estimate of 1.2 million deaths among HIV-negative people and approximately 208,000 deaths among HIV-positive people [4]. The global incidence of active TB disease is heterogeneously distributed, with low-to-middle income countries, mostly in Africa and Southeast Asia, exhibiting higher rates of TB [4].

Despite all efforts to fight this disease, the increasing emergence of multidrug-resistant strains of *Mtb*, renders standard treatment inefficient and emphasizes the urgency to develop new targeted therapeutic approaches [5,6]. According to the WHO, in 2019 approximately 500,000 people were infected with *Mtb* strains resistant to rifampicin, the most effective first-line antimycobacterial antibiotic. Of these, 78% were multi-drug resistant (MDR-TB), i.e., resistant to both rifampicin and isoniazid [4,6]. Additionally, extensively drug-resistant TB (XDR-TB), a more severe form of MDR-TB, has emerged. XDR-TB strains are resistant to isoniazid and rifampicin in addition to being resistant to a fluoroquinolone, as well as to at least one of the three second-line injectables currently available (amikacin, capreomycin, and kanamycin) [6]. In 2019, 12,350 cases of XDR-TB were notified [4]. Thus, antimicrobial resistance poses a serious threat to public health and requires immediate action. Within this context, antimicrobial peptides (AMP), have been coined as promising candidates for the treatment of antimicrobial infections, including TB [7,8,9].

### 1.2. Pathogenesis

*Mtb* enters the lungs via inhalation of aerosols. Following the entry into alveolar macrophages through receptor-mediated phagocytosis, *Mtb* arrests the acidification process of the phagosome and prevents its fusion with the lysosome, avoiding contact with potentially harmful lysosomal hydrolases, ensuring its survival [10,11]. Afterwards, *Mtb* invades the lung interstitial tissue, and dendritic cells or inflammatory monocytes transport the bacteria to the pulmonary lymph nodes for T cell priming, leading to recruitment of T and B cells to the lung parenchyma [12,13]. This promotes the release of several chemokines and cytokines, as well as the recruitment of various immune cells such as lymphocytes, which will arrange themselves in a globular structure with infected macrophages in the middle, a granuloma. A hallmark of tuberculosis, the granuloma is a double-edged sword. While it enables the host to retain the mycobacterial infection in a controlled microenvironment, it also provides the mycobacteria with an environment where it can survive through modulation of the immune response [14]. Furthermore, near its center, mature granulomas develop a hypoxic environment, which will induce the mycobacteria into a dormant state—a low metabolic activity state where the bacteria do not replicate and present a phenotypic drug resistance [15,16]. In many cases, the infection remains in a latent state. However, due to various genetics and environmental reasons (such as HIV infection), this latent infection can evolve into active TB, weeks or decades later. In this case, the granuloma develops a necrotic zone in its center, allowing the bacteria to exit and spread to other parts of the lungs and transmitting itself to other individuals via aerosols [10,14].

### 1.3. Challenges to the Standard Treatment

Mycobacteria feature a characteristic unique and complex cell wall which renders it highly hydrophobic. Its cell envelope presents an innermost layer of peptidoglycan, composed of N-glycolylmuramic acid instead of N-acetylmuramic acid, which, in turn, is linked to a layer of arabinogalactan via a phosphodiester bridge. The presence mycolic acids, unique long-chain fatty acids ranging between 60 to 90 carbons covalently linked to arabinogalactan confers high hydrophobicity and decreased permeability to anti-TB compounds, especially when considering that lipids constitute up to 60% of the cell-wall weight [17,18].

Thus far, the only available vaccine against tuberculosis is the Bacillus Calmette-Guérin (BCG), a live attenuated strain of *M. bovis*. However, it is not as efficient against adult forms of disease as it is in earlier ages i.e., infancy and school-age [19]. Standard treatment for TB comprises a multi-drug combination of isoniazid, rifampicin, and pyrazinamide. These three antibiotics are the basis of modern regimens [20]. Isoniazid is responsible for inhibiting the biosynthesis of mycolic acids, a major constituent of the mycobacterial cell wall [21], rifampicin inhibits RNA synthesis [22] and pyrazinamide disrupts the plasma membrane and energy metabolism [23]. During an initial two-month intensive stage, all three drugs are administered together with ethambutol, which inhibits arabinosyltransferases, a major key player in the synthesis of the structural component of the mycobacterial cell wall, arabinogalactan [20,21]. Afterwards, a four-month continuous administration of rifampicin and isoniazid is ensued. Even though these antibiotics are highly active towards actively replicating bacteria, the presence of dormant bacteria renders the treatment inefficient, requiring a prolonged drug administration, which often contributes to the development of MDR strains [24].

Treatment of MDR-TB is far more challenging, as it is more toxic, more expensive, and often unsuccessful [25]. Regimens vary from patient to patient, but they should include three drugs from group A of antibiotics (levofloxacin or moxifloxacin, bedaquiline or linezolid) and one of group B (clofazimine, cycloserine or terizidone). This means that the treatment needs to include at least four effective drugs. If the isolate shows resistance to any of these drugs, then additions from group C can be used (ethambutol, delamanid, pyrazinamide, imipenem-cilastarin or meropenem, amikacin or streptomycin, ethionamide or prothionamide and *para*-aminosalicylic acid). Much like the treatment regimen, the duration can vary from patient to patient, taking up to 20 months in some cases [26].

## 2. Antimicrobial Peptides as a New Tool to Tackle Antibiotic-Resistant Infections

Antimicrobial peptides (AMP, also known as host defense peptides) are essential components of the innate immune system and possess broad-spectrum activities such as antimicrobial, wound-healing, immunomodulatory and antibiofilm. They have shown to be promising drug candidates for the treatment of microbial infections, either as a monotherapy or in conjugation with other pre-existing drugs [27,28,29,30].

AMP are characterized by a short primary structure (up to 50–60 amino acid residues) and are commonly unstructured in aqueous solution [27]. However, in the presence of biological or mimetic membranes, they display remarkable structural plasticity on a very important feature, an amphipathic conformation [31,32]. This amphipathic nature that AMP possess allows interactions in both aqueous and lipid-rich environments, such as those verified in bacterial membranes [33]. Moreover, AMP are commonly cationic, presenting an overall charge of +2 to +9 [32]. Peptides with a higher charge typically display higher activity values. However, this is usually associated with high hemolysis and host toxicity. The overall positive charge allows the peptide to establish initial electrostatic interactions with the bacterial membrane, which has an overall negative charge, being one of the main drivers for peptide selectivity [34,35,36]. While bacterial membranes have their outer leaflet negatively charged, mammals have essentially zwitterionic phospholipids. This structural difference between the two types of membranes allows for the peptides to exert their activity preferentially on bacterial cells and not on our own [37].

Various conformations have been described in folded AMP such as α-helical, β-sheet and extended structures. Larger AMP can also be circular, as their backbone is cyclized. Nonetheless, these are much less flexible [38]. α-Helical AMP are a class of linear peptides known to have a broad spectrum of antimicrobial activity and a high prevalence of lysine and arginine residues, with a significant portion of hydrophobic residues (50% or more) [39]. β-Sheet AMP are amphipathic molecules stabilized by disulfide bridges and these peptides present a much higher conformational rigidity than their α-helical counterparts [40]. Together, α-helices and β-sheets are the most common secondary structures found amongst all AMP. Extended AMP are a more heterogeneous group of peptides rich in certain amino acids, specifically proline, tryptophan, and arginine [41].

**Figure 1:**
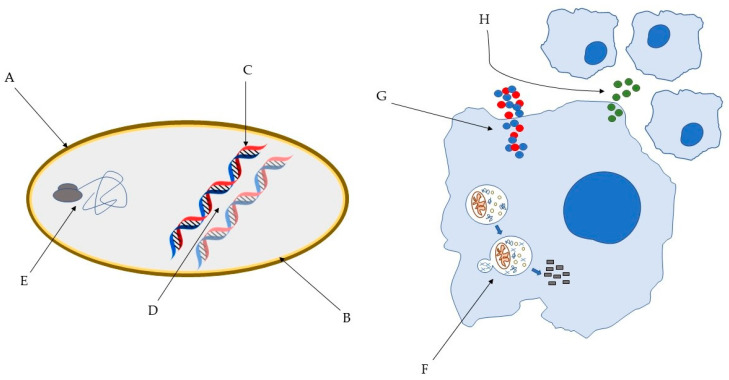
Some of the bacterial molecular targets of AMP: (**A**) bacterial cell wall; (**B**) bacterial membrane; (**C**) DNA synthesis; (**D**) DNA replication; (**E**) key bacterial protein synthesis. Some of the effects of AMP on host immune cells: (**F**) induction of autophagy on infected cells; (**G**) Overexpression of pro- and anti-inflammatory cytokines; (**H**) Chemotaxis’ induction [45,46].

Several models have been proposed to describe the pathways through which AMP exert their antimicrobial action [32] and it is well known that AMP can act through different mechanisms (Figure 1). This has a major impact on their high efficacy and the broad spectrum of activity, including Gram-positive and Gram-negative bacteria, but also fungi, viruses, unicellular protozoa, and cancer cells [41]. For instance, AMP can disrupt the membrane through pore and micelles formation, binding with specific receptors or through electroporation. Moreover, they can induce cell death through interaction with internal cellular components, hijacking important processes vital to the pathogens. They can also interfere with the cell wall through inhibition of its biosynthesis. Lastly, they are capable of exerting antimicrobial activity without ever interacting with the pathogens, through modulation of the innate immune system [32,41,42,43,44]. Remarkably, the paradigm of how peptides act is in constant change. It is now clear that several peptides are capable of exerting activity through various mechanisms. Importantly, this may hinder the development of anti-AMP resistance.

Taking into consideration the increasing resistance of *Mtb* towards conventional antibiotics and the AMP’s broad spectrum of properties, we think these molecules should be further explored as potential anti-tubercular tools. In the next sections, we will review animal, non-animal and synthetic AMP with reported anti-tubercular activity and potential for clinical use.

## 3. Animal AMP with Anti-Tubercular Activity

### 3.1. Cathelicidins

Cathelicidins are a family of mammalian AMP with approximately 30 identified members. However, in humans, rhesus monkeys, rats, mice, and guinea pigs, only a single cathelicidin is expressed, named LL-37, RL-37, rCRAMP, mCRAMP, and CAP11, respectively in each species. The peptide is produced in different cells, most notably in neutrophils, in response to an infection [47].

The expression of the human CAMP gene translates into the human cationic antimicrobial peptide-18 (hCAP18). Composed by a highly conserved *N*-terminal sequence, the cathelin, and a *C*-terminal sequence called LL-37, this propeptide is cleaved extracellularly with the *C*-terminal portion possessing bactericidal activities [48,49]. The amphipathic α-helical peptide, LL-37 is capable of binding to the negatively charged outer leaflet of the bacterial membrane, causing its disruption [50]. Moreover, LL-37 acts as a modulator of immune processes, such as chemotaxis, macrophage differentiation, neutrophils’ physiological function and apoptosis [51].

During *Mtb* infection, LL-37 was shown to be expressed after the upregulation of vitamin D receptor resulting from the interaction of mycobacterial lipopeptide with Toll-like receptor (TLR)-2 [52]. Concomitantly, Mily et al. demonstrated that the oral administration of phenylbutyrate and vitamin D3 was able to induce the expression of LL-37 in macrophages and lymphocytes, with a consequent decrease in the intracellular load of *Mtb* [53]. TLR-9 activation with *Mtb* DNA, was also correlated with high LL-37 production in alveolar macrophages [54]. This induction of LL-37 has been shown to happen 18 h after infection in lung epithelial cells, in a dose-dependent manner, with neutrophils also being capable of efficiently producing LL-37 [54]. Albeit more resistant than other mycobacterial species, *Mtb* proved to be susceptible in vitro to LL-37, with 39% killing with 25 μg/mL after 24 h and a 60% killing with 100 μg/mL after 72 h [49], demonstrating a MIC of ~5 μg/mL against *Mtb* H37Rv (Table 1) [55]. LL-37 also has immunomodulatory activities in *Mtb* infected macrophages, being capable of increasing IL-1β mRNA expression. It was also capable on increasing TNF-α levels after 4 h of peptide treatment. However, the effect of the peptide on TNF-α is time-dependent, as its levels decreased with longer peptide treatments (8 h and 24 h) at any of the concentrations tested (1, 5 and 15 μg). This indicates not only a proinflammatory role but also an immunosuppressing one. In fact, IL-10 and TGF-β levels were substantially increased in infected macrophages after stimulation with LL-37, suggesting an anti-inflammatory role to LL-37, highlighting the modulation of a balanced response orchestrated by this AMP [56]. Of note, LL-37 was not found in tuberculous granulomas, questioning its role in later phases of infection [54]. It was also verified that mCRAMP was marginally more active than LL-37 against *Mtb* H37Rv (Table 1), and its action in vivo resulted in a non-significant decreased lung area affected by pneumonia while LL-37 showed a non-significant increase [55].

Compared to other mammals, pigs have a large reservoir of cathelicidins, for instance, the linear proline-rich PR-39 [57]. Originally isolated from the porcine small intestine, it has also been discovered in bone marrow and neutrophils [58,59]. Its mechanism of action against *Escherichia coli* consists of DNA and protein synthesis’s inhibition [60]. PR-39 displayed a growth inhibition of 30% at 6.25 μg/mL and 80% at 50 μg/mL against *Mtb* H37Rv presenting an IC50 of 17 μg/mL. Two drug-susceptible clinical isolates were as susceptible as the H37Rv strain. Against an MDR strain (E1380/94), the IC50 is 93 μg/mL, with a growth inhibition of almost 50% when treated with 100 μg/mL. (Table 1) [61].

Additionally, a bovine cathelicidin named indolicidin has been recently tested against *Mtb*. Indolicidin is a 13 amino acids long tryptophan-rich peptide, expressed in neutrophils’ granules, that interacts with bacterial DNA and RNA [47]. Indolicidin was capable of exerting antimycobacterial effect against three different isolates, presenting MICs of 32 against two of them and of 64 μg/mL against the other (Table 1), while being ineffective against the remaining isolates [62].

### 3.2. Human Defensins

Defensins are small cationic peptides with broad-spectrum activity against bacteria, viruses, and fungi, predominantly expressed in epithelial cells and neutrophils. They inhibit bacterial growth through various mechanisms, depending on the defensin itself and its target. Nonetheless, direct cell membrane disruption and targeting of DNA are two pathways commonly observed. They also exert antibacterial activities through neutralization of secreted toxins and are responsible for chemotaxis [63,64,65]. Characterized by the presence of a β-sheet core structure that is stabilized by six disulphide-linked cysteines, mammalian defensins are classified into three different subfamilies that differ in the position of the disulphide bonds: alpha, beta, and theta. However, the latter is not present in humans [66]. There are six known α-defensins present in humans, four human neutrophil peptides (HNP1-4) mainly expressed by granulocytes, while the other two (HD-5 and 6) are expressed by intestinal Paneth cells. Human β-defensins (HBD) are produced by epithelial cells and several have been identified through in silico analysis [64,67].

During *Mtb* infection of alveolar epithelial cells, the expression of HBD2 was found to be upregulated [68]. Moreover, administration of L-isoleucine in murine models of TB induced a significant increase of HBD3 and HBD4, which was correlated with decreased bacterial load and tissue damage. This was observed not only in animals infected with *Mtb* H37Rv but also in those with an MDR strain [69]. Fattorini et al. reported that HBD1 inhibited the in vitro growth of both drug-susceptible H37Rv and MDR RM22 strains of *Mtb*. Furthermore, the combination of HBD1 with isoniazid significantly reduced the in vitro growth of both strains in comparison with the peptides or isoniazid alone [70]. Upregulation of α-defensins, more specifically HNP1-3, has also been linked with *Mtb* infection, with TB patients demonstrating higher levels of defensins HNP1-3 in plasma and bronchoalveolar fluid. Furthermore, a differentiated expression of HNP4 in comparison with latently *M. tuberculosis*-infected individuals has been shown [71,72]. Sharma et al. demonstrated that injection of HNP1 resulted in significant clearance of bacilli from lungs, liver and spleen of *Mtb*-infected mice [73]. Additionally, the concomitant administration of HNP and HBD with conventional anti-tubercular drugs resulted in a synergistic effect, suggesting that these peptides can be used as co-adjuvants to reduce drug dose and side effects, and possibly counteract resistance development [74]. Recently, a 15-amino acid long fragment of HNP1, Pep-H, was tested in vitro against *Mtb* H37Rv, displaying an inhibitory activity of 60% and 92% at 5 μg/mL and 10 μg/mL, respectively, the latter being taken as its MIC (Table 1). The peptide was also tested against intramacrophagic *Mtb*, demonstrating a 91% reduction of intracellular mycobacterial growth at 5 μg/mL [75]. Moreover, Pep-H not only led to a significant increase in the levels of RNOS and IFN-γ but also decreases pro-inflammatory cytokines such as TNF-α, IL-6 and MCP-1, highlighting immunomodulatory properties in addition to direct antimicrobial activity against *Mtb* [75].

### 3.3. Protegrins

The protegrins family is composed of five native AMP sequences identified in porcine leukocytes (PG-1 to PG-5) [76]. These cationic peptides are 16 to 18 amino acids long, and adopt amphipathic β-sheet structure [77,78]. Against axenically growing *Mtb* H37Rv, PG-1 displayed a 68.4% CFU reduction at 64 μg/mL and 96.7% 128 μg/mL, whereas against an MDR strain a significant decrease is only achieved at 128 μg/mL (Table 1). Nonetheless, the peptide displayed a synergistic effect when administered alongside isoniazid [70].

### 3.4. Hepcidin

Hepcidin is an AMP involved in iron homeostasis. Iron is extremely important for all living organisms, including bacteria, participating in major biological processes such as gene regulation and DNA biosynthesis [79]. It is also required to produce superoxide dismutase which protects them from the hosts’ oxygen radicals [80]. Hepcidin is composed of two short β-strands that adopt a hairpin loop and is synthesized in hepatocytes in response to an infectious or inflammatory process. Responsible for negatively regulating intestinal iron absorption and macrophagic iron release, it downregulates the transport of iron through interaction with ferroportin, responsible for exporting iron to the extracellular space. This trapping of iron inside macrophages, hepatocytes and enterocytes results in disadvantageous invasion conditions for the bacteria [80,81,82]. Sow et al. showed that hepcidin expression is upregulated in macrophage’s phagosomes following *Mtb* infection, inhibiting the growth through iron sequestering [83]. However, in a murine model of *Mtb* infection, liver hepcidin expression was found to be downregulated and its deficiency did not have a significant impact on the infection outcome [84].The possible role of hepcidin in host defense against *Mtb* is thus not clear.

### 3.5. Lactoferrin

Lactoferrin (LF) is an 80 kDa iron-binding glycoprotein present in various mammalian secretions, such as saliva, tears, and milk. Its affinity for iron is 300 times higher than serum transferrin [85]. Possessing a wide array of physiological functions such as antimicrobial and immunomodulatory, it plays a significant role in the innate immune system, being associated with host defence against oral pathogens, given its presence in saliva. Several peptides with antimicrobial activities are produced by the action of proteases on LF [86]. One of those peptides, hLF(1-11), is under clinical trials to treat infections caused by *Klebsiella pneumoniae, Listeria monocytogenes* and methicillin-resistant *Staphylococcus aureus* [87]. LFcin17-30 is another LF-derived peptide with significant activity against other mycobacteria, such as *Mycobacterium avium* [88,89]. However, to the best of our knowledge, there are no reports on the activity of this peptide against *Mtb*.

Capable of effectively interacting with membranes [90], lactoferrin was shown to be active against *Mtb* through modulation of the immune system. Using an aerosol model of infection with *Mtb* Erdman, Welsh et al. verified that mice treated with oral LF displayed a decreased bacterial dissemination to the liver and lower CFU count in the lung, accompanied by an increase in certain proinflammatory mediators, such as IL-12 [91]. Moreover, splenocytes stimulated with lipopolysaccharide (LPS) in the presence of LF increased the IL-12/IL-10 ratio, which results in the development of Th1 response, offering protection against *Mtb* [92]. Moreover, LF improves BCG-vaccine efficacy when used as an adjuvant agent, thus reinforcing the anti-tubercular potential of this protein [93,94]. However, *Mtb* is reported to be able to acquire iron through internalization of LF via GAPDH, a single receptor able to acquire iron from both transferrin and lactoferrin [95].

### 3.6. Ub2—A Ubiquitin-Derived Peptide

Ubiquitin is a protein that regulates proteasomal degradation, marking its target proteins to be destroyed in the 26S proteasome [96]. Moreover, ubiquitin is responsible for regulating the trafficking of proteins in the endocytic pathway [97]. During an infection, the fusion of the phagosome with the lysosome facilitates the killing of the invading pathogen through both oxidative and non-oxidative mechanisms. Ubiquitin-derived peptides are the primary mediators of those non-oxidative mechanisms [98]. Ubiquitin itself has no antimycobacterial activity in lysosomal extracts. However, some of its derived peptides produced by proteolytic degradation of ubiquitin, namely Ub2, are active [99].

Against *Mtb*, Ub2 possesses a MIC of 5 μM (Table 1) [99]. The mechanism through which this peptide acts depends on the formation of a secondary structure involving a β-sheet. Ub2 inserts into the bacterial membranes, being capable of disrupting it and releasing the internal content [100]. Being a short peptide, Ub2 is likely unable to span the membrane, thus it most likely acts via the micellar aggregate “detergent-like” model [101]. Nonetheless, Ub2 has also been shown to localize in the bacterial cytoplasm [100].

### 3.7. Hcl2

Hcl2 is a fragment of the human mitochondrial protein COX3. It was reported to strongly bind ESAT-6 [102], a protein secreted by *Mtb* that plays a key role in the mycobacterial pathogenesis, by suppressing the antigen presentation of macrophages [103].

Samuchiwal et al. demonstrated that 15 µg/mL Hcl2 significantly inhibited the in vitro growth of *Mtb* H37Rv by disrupting the heterodimeric ESAT-6:CFP10 complex. Moreover, a drastic reduction of the mycobacterial load in THP-1 macrophages was verified, indicating that the peptide has both extracellular and intracellular activities, while at the same time setting up a strong immune response [102].

### 3.8. Cathepsin G-Derived Peptides

Cathepsin G (catG) is a neutrophil serine protease (NSP) with antimicrobial properties. Stored within the acidic granules, NSP become active only after being released into the phagocytic vacuole. Furthermore, they are also components of neutrophil extracellular traps—extracellular fibrillary structures released by neutrophils. These traps are composed of NSP alongside chromatin and facilitate pathogen arrest [104,105].

CatG demonstrated low activity against *Mtb* Erdman, in a 24 h incubation assay [106]. At 10 μg/mL, the mycobacterial viability was reduced by 10% while at 50 to 100 μg/mL a decrease of 20 to 30% of viability was verified. On the other hand, CG117-136, a catG-derived peptide and C12-CG 117-136, a fatty acid-modified version, displayed higher activities, reaching 58% decrease at 100 μg/mL and more than 60% decrease at 10 μg/mL, respectively. Of note, the expression and activity of catG was downregulated in THP-1 monocytes exposed to the *Mtb* bacilli or LPS [106].

### 3.9. Venom-Derived Peptides

Natural toxins can be used therapeutically against several diseases, due to their high specificity for certain cellular components. Scorpion venom has been studied as a source of those toxins, as it is a mixture of polypeptides, nucleotides, mucoproteins among other substances [107]. Their AMP share relevant characteristics with the ones we are typically familiar with, such as the presence of hydrophobic and cationic residues, a positive net charge, and the ability to adopt an amphipathic structure [108].

Two AMP, VpAmp1.0 and VpAmp2.0, and their respective derivates, VpAmp1.1 and VpAmp2.1, from the scorpion *Vaejovis punctatus*, were active against *Mtb* H37Rv, presenting MICs of 17.4 μM, 5.4 μM, 21.4 μM, 13.6 μM, respectively. They were even more active against an MDR strain (except VpAmp2.0), presenting MICs of 4.2 μM, 8.6 μM, 30.5 μM and 8.5 μM, respectively (Table 1) [108]. Moreover, Pin2, a 24-amino acid long α-helical AMP extracted from the venom of the African scorpion *Pandinus imperator*, is anti-tubercular as well, presenting a MIC of 22.1 μM against *Mtb* H37Rv and 33.1 μM against an MDR strain. Two variants of Pin2, Pin2 G and Pin2 GPG were also tested, alongside two short variants of Pin2 with 14 and 17 amino acids. The 14-amino acid long short variant of Pin2 was the best peptide, with a MIC of 11.92 μM against H37Rv strain and 6 μM against an MDR strain (Table 1) [109].

Much like scorpion venom, snake venom has also been the target of research for finding new antibacterial compounds, as it is rich in biologically active compounds. Xie et al. showed that Vgf-1, a peptide containing three pairs of disulphide-bonds, was active against several strains of MDR *Mtb* with a MIC of 8.5 μg/mL (Table 1) [110].

### 3.10. B1CTcu5

B1CTcu5 is a 21-amino acids long AMP that belongs to the Brevinin-1 family, sharing a very similar sequence to its parental peptide. Brevinins are known for their antimicrobial activities against Gram-positive and Gram-negative bacteria. This cationic peptide was isolated from the skin secretion of the frog *Clinotarsus curtipes* [111,112].

Against planktonic cultures of *Mtb* H37Rv this peptide was bactericidal, presenting a MIC of 12.5 μg/mL (Table 1). Moreover, at its MIC, the peptide was capable of completely inhibiting *Mtb* growing inside THP1-derived macrophages. Furthermore, it was deemed as non-toxic against this cell line, proving to be a potential anti-tubercular lead [112].

**Table 1:** Animal AMP covered in this review, alongside their sequence, reported direct activity, and respective source.

Peptide	Sequence	Activity	Source	Ref.
LL-37	LLGDFFRKSKEKIGKEFKRIVQRIKDFLRNLVPRTES	MIC (H37Rv): ~5 μg/mL	Various human immune cells	[55]
mCRAMP	GLLRKGGEKIGEKLKKIGQKIKNFFQKLVPQPEQ	MIC (H37Rv)—<5 μg/mL	Various mice immune cells	[55]
PR-39	RRRPRPPYLPRPRPPPFFPPRLPPRIPPGFPPRFPPRFP	IC_50_ (H37Rv): 17 μg/mL IC_50_ (MDR E1380/94): 93 μg/mL	Porcine small intestine, bone marrow, neutrophils	[61]
Indolicidin	ILPWKWPWWPWRR	MIC (*Mtb* clinical isolate): 32 μg/mL	Bovine neutrophils’ granules	[62]
Pep-H	RRYGTCIYQGRLWAF	MIC (H37Rv): 10 μg/mL	HNP-1 in human granulocytes	[75]
PG-1	RGGRLCYCRRRFCVCVGR	68.4% CFU reduction at 64 μg/mL	Porcine leukocytes	[70]
Ub2	STLHLVLRLRGG	MIC (CDC1551): 5 μM	Ubiquitin in lysosomal extracts	[99]
VpAmp1.0	LPFFLLSLIPSAISAIKKI, amidated	MIC (H37Rv): 17.4 μM MIC (MDR clinical isolate): 4.2 μM	*Vaejovis punctatus* venom	[108]
VpAmp2.0	FWGFLGKLAMKAVPSLIGGNKSSSK	MIC (H37Rv): 5.4 μM MIC (MDR clinical isolate): 8.6 μM	*Vaejovis punctatus* venom	[108]
VpAmp1.1	FFLLSLIPSAISAIKKI, amidated	MIC (H37Rv): 21.4 μM MIC (MDR clinical isolate): 30.5 μM	*Vaejovis punctatus* venom	[108]
VpAmp2.1	FWGFLGKLAMKAVPSLIGGNKK	MIC (H37Rv): 13.6 μM MIC (MDR clinical isolate): 8.5 μM	*Vaejovis punctatus* venom	[108]
Pin2	FWGALAKGALKLIPSLFSSFSKKD	MIC (H37Rv): 22.1 μM MIC (MDR clinical isolate): 33.1 μM	*Pandinus imperator* venom	[109]
Pin2[14]	FWGLKGLKKFSKKL	MIC (H37Rv): 11.92 μM MIC (MDR clinical isolate): 6 μM	*Pandinus imperator* venom	[109]
Vgf-1	ECYRKSDIVTCEPWQKFCYREVTFFPNHPVYLSGCASECTETNSKWCCTTDKCNRARGG	MIC (MDR *): 8.5 μg/mL	*Naja atra* venom	[110]
BICTcu5	LIAGLAANFLPQILCKIARKC	MIC (H37Rv): 12.5 μg/mL	*Clinotarsus curtipes* skin secretion	[112]

* Various MDR clinically isolated strains resistant to different antibiotics.

## 4. Non-Animal AMPs with Anti-Tubercular Activity

### 4.1. Bacterial Peptides

#### 4.1.1. Nisin A and Lacticin 3147

Nisin A and lacticin 3147 are two of the best characterized lantibiotics. Ribosomally synthesized peptides produced by the Gram-positive bacteria *Lactococcus lactis*, lantibiotics have had their use well-documented in food, animal, and human applications [113]. Nisin A is a 34-amino acids long peptide with reported activity against several bacteria, including mycobacteria. It acts on the cytoplasmic membrane through pore formation and through interruption of cell wall biosynthesis after establishing contact with the lipid II molecule [114]. Lacticin 3147, much like nisin, is active against many drug-resistant bacteria, including clinically relevant mycobacteria. It is a “two-peptide lantibiotic” since it acts through the synergistic action of Ltnα and Ltnβ, 30 and 29 amino acids in length, respectively. It acts via the formation of pores that are selective to K^+^ ions and inorganic phosphate, which leads to the dissipation of the membrane potential and hydrolysis of internal ATP, culminating in cell death through the collapse of the pH gradient [115].

Carrol et al. reported that both nisin A and lacticin 3147 are active in vitro against *Mtb*. Lacticin 3147 presented an IC_90_ of 7.5 μg/mL against *Mtb* H37Ra (Table 2). This peptide displayed a 98% growth inhibition at 60 μg/mL, while nisin A could only inhibit 76.3% of the culture growth at the same concentration (Table 2) [116]. However, it has been demonstrated that mutated variants of nisin, nisin K22S (nisin S), nisin K22T (nisin T) and nisin M21V (nisin V) were more potent than nisin A against *Mtb* H37Ra. Nisin V was 10% more potent, while nisin T and nisin S reduced the mycobacterial growth by 24% and 26%, respectively, when compared to nisin A (Table 2) [117].

#### 4.1.2. Mutacin 1140

Mutacin 1140, typically known as MU1140, is derived from *Streptococcus mutans* JH1140, has 22 amino acids and presents low levels of toxicity, a high degree of stability, and good pharmacokinetics [118,119]. Its mode of action is thought to be similar to nisin, as it is believed that MU1140 interacts with lipid II and consequently inhibits cell wall synthesis [120]. Noteworthy, MU1140 is an Oragenics Inc. (Tampa, FL, USA) lead compound, successfully concluding pre-clinical trials after displaying promising activity against both active and latent *Mtb* [8].

#### 4.1.3. Lasso Peptides

Lasso peptides are a subclass of ribosomally synthesized and post-translationally modified peptides characterized by a stable structure. With a broad spectrum of bioactivity, lasso peptides have been associated with antimicrobial action, including relevant anti-tubercular efficacy [121].

Lassomycin is a peptide that belongs to the subclass of lasso peptides and it is extracted from *Lentzea kentuckyensis* sp. It displays potent activities against various strains of *Mtb,* including MDR and XDR strains, ranging from 0.8 to 3 μg/mL (Table 2) [122]. It acts by targeting the ATP-dependent protease ClpC1P1P2, an important regulatory enzyme that destroys key cellular proteins. The inhibition of this proteolysis is a novel mechanism through which a peptide acts and it is highly detrimental to bacteria, as proven by this peptide’s anti-tubercular activity [122].

Lariatins A and B, both derived from *Rhodococcus jostii* K01-B0171, are other AMP that belong to the lasso peptides subclass and have reported anti-mycobacterial activities [123]. Even though both lariatin A and B are active against *M. smegmatis*, only lariatin A was tested against *Mtb* H37Rv in vitro, displaying a MIC of 0.39 μg/mL (Table 2) [123].

#### 4.1.4. Streptomyces-Derived Peptides

Ever since the description of S-520’s activity in the 1970s, *Streptomyces* has been a bacterial source of peptides with antimycobacterial properties [124]. Most importantly, these bacteria are the source of streptomycin, the first approved antibiotic used to treat tuberculosis [125].

More recently, some AMP originated from *Streptomyces* have been identified as anti-tubercular. An actinomycete extract conveyed identification of hytramycins I and V, two cyclic peptides that originated from *Streptomyces hygroscopicus*. These peptides displayed potent in vitro activities against *Mtb* H37Rv, with MICs of 6 and 11.3 μg/mL under normal conditions and MICs of 1.5 and 2.4 μg/mL under hypoxic conditions, respectively (Table 2). Hytramycin I was further shown to present MICs lower than 10 μg/mL against 7 *Mtb* MDR strains [126].

Cyclomarin A is another cyclic AMP that exhibited activity against several *Mtb* MDR isolates, killing more than 90% of nonreplicating hypoxic mycobacteria at 2.5 μM (Table 2). It was found to act through targeting of the ClpC1 subunit of the caseinolytic protease, a regulatory subunit [127]. Sansanmycin A, an AMP that belongs to the novel class of uridyl-peptide antibiotics that originate from *Streptomyces* sp. SS, presented a MIC of 16 μg/mL against *Mtb* H37Rv, and 8 μg/mL against *Mtb* 2199, a strain isolated from patients in China resistant to both rifampicin and isoniazid (Table 2) [128].

### 4.2. Fungal Peptides

#### 4.2.1. NZX

Fungal defensins are potent AMP with low toxicity and high serum stability. Plecstasin was the first fungal defensin identified. Derived from the fungus *Pseudoplectania nigrella,* it is a 40-amino acid long peptide with described activity against Gram-positive bacteria, including MDR strains, such as *Staphylococcus aureus.* Through the method of residue substitution, several variants of plecstasin were generated such as NZX, with reported anti-tubercular activities and high resistance to proteases’ action [129].

NZX demonstrated a concentration-dependent activity, with a MIC of 6.3 μM against *Mtb* H37Rv and an MDR isolate. Against two clinical isolates, it displayed MICs of 6.3 μM and 3.2 μM, with 6.3 μM being also the MIC obtained against an MDR isolate (Table 2) [129]. In vivo, an intra-tracheal administration of 0.83 mg of NZX during 5 days, after a 19-day infection of BALB/c mice with *Mtb*, reduced the number of CFUs present in the lungs by 46% compared to the control animals. These results are comparable to the effects of rifampicin, which strongly supports the peptide’s therapeutic potential as a future anti-tubercular agent [129].

#### 4.2.2. Trichoderins

In 2010, using a screening system, Pruksakorn et al. were able to isolate three new aminolipopeptides named trichoderins A, A1 and B. These were isolated from a culture of marine sponge-derived fungus of *Trichoderma* sp. 05FI48 with the premise of being AMP with anti-dormant mycobacterial properties [130].

Using both aerobic and hypoxic conditions, Pruksakorn et al. were able to assess that all three trichoderins had the same activity against *Mtb* H37Rv in both conditions, with MICs of 0.12 μg/mL, 2 μg/mL and 0.13 μg/mL for trichoderin A, A1 and B, respectively (Table 2) [130]. Trichoderin A, the peptide with the highest efficacy, was found to exert this potent antimycobacterial activity through inhibition of the mycobacterial ATP synthesis [131].

### 4.3. Plant-Derived Peptides

#### Capsicum Defensins

As mentioned before, many AMP are part of an innate response elicited by most living forms, and plants are not an exception. *Capsicum* plants, specifically, have been considered as a possible research target in the search for new proteins and peptides that help defend plants against invading pathogens, given their already described antibacterial compounds [132,133].

Gebara et al. identified two fractions from an isolate of pepper fruits of *Capsicum annuum* with antimycobacterial activity. These two fractions demonstrated two protein bands at ~5 and ~6 kDa. They were later characterized as the result of two defensins—*Ca*Def_2.1_ and *Ca*Def_2.2_ [134]. Fraction F2, containing *Ca*Def_2.1_ and *Ca*Def_2.2_, demonstrated an IC_50_ of 39.2 μg/mL against *Mtb* H37Rv (Table 2). F3, possessing *Ca*Def_2.2_, was not able to maintain its potency, demonstrating an IC_50_ > 100 μg/mL. However, against the hypervirulent *Mtb* M299 strain, no fraction could present an IC_50_ < 100 μg/mL [134].

**Table 2:** Non-animal AMP covered in this review, alongside their sequence, reported direct activity, and respective source.

Peptide	Sequence	Activity	Source	Ref.
Nisin A	I-Dhb-AI-Dha-LA-Abu-PGAK-Abu-GALMGANMK-Abu-A-Abu-ANASIHV-Dha-K	IC_90_ (H37Ra): >60 μg/mL	*Lactococcus lactis*	[116]
Nisin S	I-Dhb-AI-Dha-LA-Abu-PGAK-Abu-GALMGANMS-Abu-A-Abu-ANASIHV-Dha-K	Mycobacterial growth reduced in 26% compared to Nisin A	Derived from Nisin A	[117]
Nisin T	I-Dhb-AI-Dha-LA-Abu-PGAK-Abu-GALMGANMT-Abu-A-Abu-ANASIHV-Dha-K	Mycobacterial growth reduced in 24% compared to Nisin A	Derived from Nisin A	[117]
Nisin V	I-Dhb-AI-Dha-LA-Abu-PGAK-Abu-GALMGANVK-Abu-A-Abu-ANASIHV-Dha-K	Mycobacterial growth reduced in 10% compared to Nisin A	Derived from Nisin A	[117]
Lacticin 3147	Equimolar mixture of Lanα [AA-Dhb-N-Dhb-F-(D-Ala)LADYWGNNGAWA-Abu-L-Abu-HEAMAWAK] and Lanβ [[CH_3_-CH_2_-CO-CO-Dhb-PA-Dhb-PAI-(D-Ala)IL-(D-Ala)AYIATNTAP-Abu-TKA-Abu-RAA]	IC_90_ (H37Ra): 7.5 μg/mL	*Lactococcus lactis*	[116]
Lassomycin	GLRRLFADQLVGRRNI-CO_2_CH_3_	MICs (MDR and XDR strains *): 0.8–3 mg/mL	*Lentzea kentuckyensis* sp.	[122]
Lariatin A	cyclo-(GSQLVYRE)-WVGHSNVIKP	MIC (H37Rv): 0.39 μg/mL	*Rhodococcus jostii* K01-B0171	[123]
Hytramycin V	cyclo-[(NMe-Ala)-(D-Pip)-L-(D-Val)-Pip-(D-Pip)]	MIC Normal (H37Rv): 11.37 μg/mL MIC Hypoxic (H37Rv): 2.4 μg/mL	*Streptomyces hygroscopicus*	[126]
Hytramycin I	cyclo-[(NMe-Ala)-(D-Pip)-L-(D-Allo-Ile)-Pip-(D-Pip)]	MIC Normal (H37Rv): 6 μg/mL MIC Hypoxic (H37Rv): 1.5 μg/mL	*Streptomyces hygroscopicus*	[126]
Cyclomarin A	cyclo-[(tert-prenylated β-hydroxy-Trp)-(NMe-5-hydroxy-Leu)-A-(β-methoxy-Phe)–V-(NMe-Leu)–(2-amino-3,5-dimethyl-4-hexeneoic acid)]	>90% of the inoculum killed in five days at 2.5 μM	*Streptomyces* sp. (CNB-982)	[127]
Sansanmycin A	Not available	MIC (H37Rv): 16 μg/mL MIC (2199): 8 μg/mL	*Streptomyces* sp. SS	[128]
NZX	GFGCNGPWSEDDIQCHNHCKSIKGYKGGYCARGGFVCKCY	MIC (H37Rv and clinical MDR isolate): 6.3 μM MIC (clinical isolates): 3.2–6.3 μM	Derived from plecstasin of *Pseudoplectania nigrella*	[129]
Trichoderin A	(MDA)-P-(AHMOD)-Aib-Aib-IL-Aib-Aib-(AMAE)	MIC (H37Rv): 0.12 μg/mL	*Trichoderma* sp. 05FI48	[130]
Trichoderin A1	(MDA)-P-(AHMOD2)-Aib-Aib-IL-Aib-Aib-(AMAE)	MIC (H37Rv): 2 μg/mL	*Trichoderma* sp. 05FI48	[130]
Trichoderin B	(MDA)-P-(AHMOD)-Aib-Aib-LL-Aib-Aib-(AMAE)	MIC (H37Rv): 0.13 μg/mL	*Trichoderma* sp. 05FI48	[130]
CaDef_2.1_	ICEALSGNFKGLCLSSRREGFTDGSCIGFRLQCFCTKPCA	MIC (H37Rv): 39.2 μg/mL	*Capsicum annuum*	[134]
CaDef_2.2_	SKYFTGLCWTDSSCRKVCIEKDFQDGHCSKIQR			

Abu: α-aminobutyric acid; AHMOD: (2*S*,4*S*,6*S*)-2-amino-6-hydroxy-4-methyl-8-oxodecanoic acid; AHMOD2: (2S,4R)-2-amino-4-methyl-8-oxodec-6-enoic acid; Aib: 2-aminoisobutyric acid; AMAE: (*S*)-2-((2-aminopropyl(methyl)amino)etanol; Dha: dehydroalanine; Dhb: dehydrobutyrine; MDA: (*R*)-2-methyldecanoic acid; Pip: piperazic acid; * various MDR and XDR strains resistant to different antibiotics.

## 5. Synthetic AMPs with Anti-Tubercular Activity

### 5.1. De Novo Designed Peptides

In the de novo design of synthetic AMPs, important characteristics such as an amphipathic conformation and a cationic net charge will be favored. However, subtle properties selected by evolution may be missing, which may reflect on different folding patterns or the lack of specificity of these AMP [135]. On the other hand, *de novo* designed non-natural peptides and peptidomimetics may exhibit improved pharmacokinetics, including higher resistance to proteolytic degradation in vivo [136].

Peptoids, also called oligo-N-substituted glycines, are peptidomimetics with a backbone identical to natural proteins but with amino acid side chains attached to the backbone nitrogen instead of the α-carbon. This confers the peptoid high resistance to proteases [137]. Kapoor et al. showed that 1-C13_4mer_, a designed tetrameric, alkylated and cationic peptoid, was highly active against *Mtb* H37Rv, presenting a MIC of 6.6 μM (Table 3) while displaying no cytotoxicity, whereas an unalkylated analogue was inactive [138].

In 2016, Hicks et al. designed de novo AMPs to overcome certain issues such as bioavailability and metabolic stability. These authors incorporated C^α^-tetra-substituted α-amino acids, known to induce stable secondary structures. Ten peptides were tested, all of them displaying a MIC of <41 μM against H37Rv, MDR, and XDR strains of *Mtb,* with the most active peptide presenting a MIC of 4.92 μM against all three strains (Table 3) [139].

M(LLKK)_2_M is another synthetic α-helical peptide designed with the primary backbone sequence (XXYY)n where X is a hydrophobic amino acid, Y is a cationic amino acid and n is the number of repeat units, which was proven to be active in vitro against *Mtb* with a MIC of 125 μg/mL against H37Rv strain and 62.5 μg/mL against MDR CSU87 strain (Table 3). When combined with rifampicin this peptide demonstrated a synergistic effect, delaying the emergent rifampicin-resistance [140].

D-LAK120 peptides are a family of six AMP composed of 25 D-amino acids with a charge angle of 120° that adopt a left-handed α-helix conformation [141]. Their activity against drug-resistant strains of *Mtb* was assessed both in vitro and ex vivo by Lan et al. [142]. All six peptides successfully inhibited the growth of *Mtb* in vitro in a concentration-dependent manner. Four of these peptides significantly inhibited the growth of *Mtb* inside THP-1 cells, measured after 7 days in culture with 6.25 μM D-LAK120 peptides. A combination of one of the peptides, D-LAK 120-A, with isoniazid potentiated the effect against a drug-resistant strain of *Mtb* [142].

Ramón-García et al. also reported anti-tubercular activities through *de novo* synthesized cationic, short, nontoxic AMP [143]. Out of 49 peptides, twelve had an IC_90_ lower than 5 μM while the most potent peptide (WKWLKKWIK) had an IC_90_ of 1 μM (Table 3) [143].

Silva et al. demonstrated that the addition of cinnamic acids to the N-terminus of seven cationic AMP enhanced their antimycobacterial potency, against *Mtb* H37Rv and a MDR strain, with Cin+CAMP1 and Cin+CAMP3 being the most potent peptides [144]. They displayed an IC_95_ of 44.33 μM and 38.51 μM, respectively, and an IC_50_ of 0.69 μM and 2.41 μM against *Mtb* H37Rv. Furthermore, against a MDR strain, Cin+CAMP1 and Cin+CAMP3 displayed an IC_50_ of 2.77 μM and 0.60 μM (Table 3) [144].

Iztli Peptide 1 (IP-1) is a synthetic hunter-killer peptide—a molecule that comprises a ligand peptide (hunter sequence) and a cationic antibacterial peptide (killer sequence) [145]. Coyotl et al. demonstrated that IP-1 was able to directly kill *Mtb* H37Rv and a clinical MDR isolate CIBIN99 in vitro with an IC_50_ of 99.27 μM and 92.66 μM, respectively (Table 3). Treating infected animals with H37Rv strain led to a significant reduction of the lung bacillary load (greater than 80%) and a slight reduction of pneumonia through an every other day intratracheal administration of 8 μg of IP-1 for one month, after two months of infection. Similar results were observed for the MDR strain. Furthermore, this peptide was able to induce autophagy in macrophages and HEK293T (a variant from the human embryonic kidney 239 cells) and induce the production of TNF-α. These data prove that this peptide might be useful in treating *Mtb* infections through induction of the protective cytokine TNF-α while at the same time inducing autophagy in infected macrophages and through direct killing of *Mtb* [146].

### 5.2. Nature-Inspired Synthetic Peptides

Rivas-Santiago et al. assessed the inhibitory activity of three synthetic peptides against *Mtb*—E2, E6 and CP26 [55]. E2, an octapeptide, and E6, a dodecapeptide, are the result of peptide array and substitution screenings on bactenecin, a bovine dodecapeptide. CP26 is a β-helical 26-amino acids long, derived from a hybrid peptide containing the amphipathic α-helical *N*-terminal region of cecropin A and the hydrophobic *N*-terminal of the bee venom melittin. These peptides were proven effective in reducing the mycobacterial growth of both susceptible (H37Rv) and MDR strains of *Mtb* with MICs of 2.1 (CP26), 2.6 (E2), and 3.2 μg/mL (E6) (Table 3) [55].

Through substitution, scrambling and screening for the capacity to induce chemokines like macrophage chemotactic protein-1 (MCP-1), three immunomodulatory IDR peptides, IDR-HH2, IDR-1002 and IDR-1018 were formulated, synthesized, and tested against *Mtb* in vitro [147]. IDR-1018 was the most efficient, with a MIC of 16 μg/mL followed by 1002 and HH2, both with a MIC of 29.3 μg/mL against *Mtb* H37Rv (Table 3). These peptides were not toxic towards monocytes in the concentrations tested (up to 128 μg/mL). Furthermore, following 60 days of infection, HH2 and IDR-1018 were able to significantly reduce by 3 to 5-fold CFU counts of MDR in infected mice through an intratracheal instillation of 32 μg in 100 μL of saline solution three times a week for up to 4 weeks, whereas 1002 showed no significant effect [147].

Li et al. not only tested the activity of sansanmycin A against *Mtb*, already mentioned above in Section 4.1.4, but also tested the activity of sansanmycin A derivatives [128]. The introduction of various substituents at the *N*-terminus was performed to increase lipophilicity and, consequently, led to an improved passive diffusion through the cytoplasmic membrane, given that its mode of action consists of inhibiting the MraY translocase present in the bacterial membrane. Out of 17 derivatives, only one was more effective than its parental peptide, the analogue 1d, possessing an isopropyl group. Against both *Mtb* H37Rv and 2199 strains, this synthetic derivative had a MIC of 8 μg/mL (Table 3) [128].

AK15-6 is an isomer of the mycobacteriophage-derived AK15 peptide, formed by a rearrangement of the amino acids of the AK15’s helix to increase its hydrophobic moment [148]. Both AK15 and its synthetic isomer showed selective toxicity towards *Mtb*, with no activity against Gram-negative and Gram-positive bacteria below 150 μg/mL. Against *Mtb* H37Rv, AK15 displayed a MIC of 37.5 μg/mL while AK15-6 demonstrated a MIC of 18.75 μg/mL (Table 3). Both peptides inhibited mycobacterial growth through membrane disruption and trehalose 6,6’-dimycolate (TDM)-binding (the most abundant glycolipid produced on the surface of mycobacteria). Furthermore, they displayed synergistic effects with rifampicin. In the lungs of *Mtb*-infected mice, an intravenous injection of 10 mg/kg of peptides once a day over 7 days post infection reduced both the bacterial load and the number and size of tuberculous granulomas 4 weeks post infection. AK15-6 treatment inhibited 79% of the mycobacterial load, 72.2% granuloma number and 67.5% granuloma size in comparison with the control (PBS-treated mice). The peptides were also shown to exhibit immunomodulatory properties with inhibition of proinflammatory response in both TDM stimulated or *Mtb*-infected bone marrow-derived macrophages and mice [148].

**Table 3:** Synthetic AMP covered in this review, alongside their sequence, reported activity, and respective source.

Peptide	Sequence	Activity	Source	Ref.
1-C13_4mer_	H-Ntridec-NLys-Nspe-Nspe-NLys, amidated	MIC (H37Rv): 6.6 μM	*De novo*	[138]
1	Ac-GF-(A6c)-G-(A6c)-KK-(A6c)-G-(A6c)-F-(A6c)-G-(A6c)-GKK-(A6c)-KKKK, amidated	MIC (H37Ra, MDR, XDR): 4.92 μM	*De novo*	[139]
M(LLKK)_2_M	MLLKKLLKKM	MIC (H37Rv): 125 μg/mL MIC (MDR CSU87): 62.5 μg/mL	*De novo*	[140]
WKWLKKWIK	WKWLKKWIK	IC_90_ (lux): 1 μM	*De novo*	[143]
Cin+CAMP1	(Cin1)-KWLKKWIK, amidated	IC_50_ (H37Rv): 0.69 μM IC_50_ (MDR *): 2.77 μM	*De novo*	[144]
Cin+CAMP3	(Cin2)-ARLWWWWRRK, amidated	IC_50_ (H37Rv): 2.41 μM IC_50_ (MDR *): 0.60 μM	*De novo*	[144]
IP-1	KFLNRFWHWLQLKPGQPMY	IC_50_ (H37Rv): 99.27 μM IC_50_ (MDR CIBIN99): 92.66 μM	*De novo*	[146]
E2	RIWVIWRR, amidated	MIC (H37Rv, MDR clinical isolate): 2.6 μg/mL	Bactenicin	[55]
E6	RRWRIVVIRVRR, amidated	MIC (H37Rv, MDR clinical isolate): 3.2 μg/mL	Bactenicin	[55]
C26	KWKSFIKKLTSAAKKVVTTAKPLISS	MIC (H37Rv, MDR clinical isolate): 2.1 μg/mL	Bactenicin	[55]
IDR-HH2	VQLRIRVAVIRA, amidated	MIC (H37Rv): 29.3 μg/mL **	MCP-1	[147]
IDR-1002	VQRWLIVWRIRK, amidated	MIC (H37Rv): 29.3 μg/mL **	MCP-1	[147]
IDR-1018	VRLIVAVRIWRR, amidated	MIC (H37Rv): 16 μg/mL **	MCP-1	[147]
Sansanmycin A derivative 1d	Not available	MIC (H37Rv, MDR 2199): 8 μg/mL	Sansanmycin A	[128]
AK15-6	AVKKLLRWWSRWWKK	MIC (H37Rv): 18.75 μg/mL	AK15	[148]

A6c: 1-aminocyclohexane carboxylic acid; Cin1: cinnamic acid; Cin2: 2,3-dimetoxycinnamic acid; *—clinical isolate resistant to isoniazid, rifampicin and streptomycin; **—immunomodulatory activity.

## 6. Taking AMPs from Bench to the Clinic

Antimicrobial peptides, given their multi-functionality, direct killing mechanism, and immunomodulatory properties, provide an attractive pharmacological option not only against *Mtb* but also a wide range of other infections. Therefore, there has been extensive efforts on exploring the possibilities that peptides bring to the table as new therapeutic agents against infectious diseases [149]. However, despite their remarkable properties, AMPs still face major challenges to join the pharmaceutical industry. The primary challenge is the susceptibility to proteolytic enzymes. When administered orally, AMP must overcome enzymes that operate through the digestive tract, like pepsin, trypsin, and chymotrypsin. Intravenous administration poses similar challenges, as there are many proteases in blood [150]. Moreover, intravenous administration translates into a shorter half-life due to hepatic and renal clearances [31]. New design strategies have been applied to overcome these challenges, with the incorporation of non-natural amino acids, backbone mimetics, conjugation with fatty acids, and *N* and *C*-terminus modifications [136]. Furthermore, drug delivery systems, using different types of vehicles such as nanoparticles, liposomes, or different gel formulations, have also been a strategy employed to reduce proteolytic degradation [151]. Nanoparticles allow for the protection of peptides, controlled plasma levels, a prolonged and/or controlled release, and reduction of administration frequency. Consequently, all these advantages translate into lower toxicity for the host [152], a major benefit, especially when considering that another challenge that AMP need to surpass is their toxicity towards eukaryotic cells [150]. Given the broad range of activities that AMP possess, continuous large-scale production is required, especially since the availability of those naturally synthesized is rather low. Large-scale synthesis of AMP has been improving, aiming at the future attainment of industrial production levels similar to current ones for small molecule-based medicines. Furthermore, biotechnological fabrication approaches have been developed to enhance the production of AMP such as insertion of recombinant DNA into specific vectors, transgenic expression in plants and the engineering of chloroplasts as bioreactors for AMP synthesis [153,154].

Tuberculosis is still, up to this day, one of the deadliest infectious diseases ever known to mankind. Facing the possibility of losing the fight due to emerging resistance, it is imperative to keep searching for new alternatives to the current treatments. To that end, due to their non-specific mode of action, synthetic and natural AMP, from animal to non-animal sources, are expected to be the next generation of wide-spectrum antimicrobials. Still, peptides are yet to meet their expectations, mainly due to pharmacokinetic liabilities. Nonetheless, their huge plethora of applications and mechanisms cement their position as the next “go-to” anti-infective agents, hopefully useful against formidable pathogens like *Mtb.*

## Data Availability

Not applicable.
